# Clinical care for severe and persistent eating disorders in pediatric populations: Perspectives of health professionals

**DOI:** 10.1186/s40337-024-01044-6

**Published:** 2024-06-17

**Authors:** Jennifer S. Coelho, Tanya Pardiwala, Sheila K. Marshall, Pei-Yoong Lam, Seena Grewal, Alice Virani, Alexandra Olmos Pérez, Josie Geller

**Affiliations:** 1 Provincial Specialized Eating Disorders Program for Children and Adolescents, BC Children’s Hospital, 4500 Oak St., Box 150, Vancouver, BC V6H3N1 Canada; 2 Department of Psychiatry, University of British Columbia, Vancouver, BC Canada; 3 School of Social Work, University of British Columbia, Vancouver, BC Canada; 4 Division of Adolescent Health and Medicine, Department of Pediatrics, University of British Columbia, Vancouver, BC Canada; 5 Provincial Health Services Authority Ethics Service, Vancouver, BC Canada; 6 Provincial Adult Tertiary and Specialized Eating Disorders Program, St. Paul’s Hospital, Vancouver, BC Canada

**Keywords:** Severe and enduring, Eating disorder, Pediatric, Health professional, Treatment allocation

## Abstract

**Objective:**

Models of treatment for adults with severe and enduring eating disorders focus on harm reduction and improving quality of life. However, there is a notable gap in the pediatric literature in this area. The current study set out to assess the perspectives of health professionals regarding clinical care for young people (e.g., ages 10–25 years) with severe and enduring eating disorders, and to explore perceptions about appropriate treatment options for these presentations.

**Methods:**

Health professionals were invited to complete a two-stage online survey about their experiences with clinical care for pediatric eating disorders through Canadian and Australian professional eating disorder networks. Survey 1 included questions about their experiences in supporting individuals with severe and enduring presentations. Participants who completed Survey 2 reviewed clinical vignettes and shared their perspectives about treatment recommendations and models of care, including for a severe and enduring presentation.

**Results:**

A total of 85 clinicians responded to questions on Survey 1 about severe and enduring eating disorder presentations. A portion of these respondents (*n* = 25) also participated in Survey 2. The majority of respondents to Survey 1 reported providing clinical care for pediatric severe and enduring eating disorder presentations. Amongst respondents to Survey 2, there was low consensus amongst respondents for the clinical care that would be most appropriate for young people with a severe and enduring eating disorder presentation. Numerous challenges in models of care for severe and enduring presentations in pediatric settings were raised in responses on Survey 2, with clinicians sharing their awareness of models focusing on quality of life, while also raising concerns about the appropriateness of these models for young people.

**Conclusions:**

The preliminary results of this study demonstrate that the majority of clinicians report that they have provided care to young people with severe and enduring presentations. There is a clear need for establishing guidance for clinicians working in pediatric eating disorder settings around models of care focused on quality of life. Engagement with interested parties, including those with lived experience, can clarify the development of terminology and clinical pathways for severe and enduring presentations of pediatric eating disorders.

**Supplementary Information:**

The online version contains supplementary material available at 10.1186/s40337-024-01044-6.

## Abstract (plain language summary)

Treatment models focusing on harm reduction and quality of life (as opposed to eating disorder recovery) are available for adults with severe and enduring eating disorders. However, these models are not widely available for young people. In fact, there is very limited research on severe and enduring eating disorder presentations in pediatric populations. We assessed the views of health professionals regarding clinical care for young people with severe and enduring eating disorder presentations, and asked professionals about what treatment options might be most appropriate for these presentations. Most participants reported providing clinical care for pediatric severe and enduring eating disorder presentations. However, clinicians had diverse views about the treatment that would be most appropriate for a severe and enduring eating disorder presentation in a young person. Further research and engagement with clinicians and those with lived experience is needed to clarify the terminology and clinical pathways for severe and enduring presentations of pediatric eating disorders.

**Supplementary Information:**

The online version contains supplementary material available at 10.1186/s40337-024-01044-6.

## Introduction

There is a growing body of literature on chronic presentations of eating disorders in adults, particularly anorexia nervosa (AN). Proposed criteria for severe and enduring AN were published to address challenges in classifying and communicating about this condition [1]. These criteria put forth a minimum duration of illness of at least 3 years, in addition to functional impairment, persistent eating disorder symptoms, and exposure to at least two evidence-based treatments [1]. However, it is noteworthy that a Delphi study of international experts demonstrated a lack of consensus on terminology for long-standing eating disorder presentations, and that participants endorsed terms that were perceived to be more neutral, including “persistent” eating disorders [2]^1^. Furthermore, the terminology of “severe and enduring” eating disorders has not been used in the pediatric eating disorder literature [4], and there are potential harms of the term “enduring” (see e.g [5]). The current study was designed to explore the perceptions and experiences of health professionals with severe and enduring eating disorder presentations in young people (i.e., ages 10–25 years).

Alternative models of care have been developed for adults with severe and enduring eating disorders. Harm reduction approaches have been proposed, primarily focused on mitigating potential harms associated with eating disorder symptoms in adults with a long history of AN [6]. The harm reduction approach focuses on offering treatment to the individual with AN to improve quality of life, as opposed to an explicit goal of eating disorder recovery. One of the early models, the Community Outreach Partnership Program (first developed in Canada in 1995), focused on harm reduction, motivational enhancement and psychosocial rehabilitation [7]. Similar alternative models of care have been developed internationally, including the United Kingdom [8], and Finland [9]. These models are active treatments which engage individuals with severe and enduring eating disorders in goal setting around quality of life. However, this literature is specific to adults with eating disorders, and there remains as key clinical question about when a quality-of-life focus would be appropriate.

The Short Treatment Allocation Tool for Eating Disorders (STATED) [10] can facilitate clinical decision-making, and guide recommendations for interventions that focus on quality of life vs. recovery. The STATED proposes tailoring treatment to an individual based on three features: medical stability, symptom severity/life interference, and readiness/engagement [10]. Readiness/engagement refers to individuals’ interest and willingness to make behavioural changes and work towards recovery [11]]. According to the STATED, a quality-of-life focus is appropriate for those who have high symptom severity and/or life interference, and low readiness/engagement. Outpatient support is provided with regular medical monitoring, with intermittent inpatient support as needed to support harm reduction goals (e.g., brief symptom interruption admissions).

An advantage of using the parameters put forth in the STATED (i.e., medical stability, symptom severity/life interference, and readiness/engagement; [10]) is that an individual does not need to be labelled as having a “severe and enduring eating disorder” (SEED) to access a clinical pathway focused on quality of life. Concerns about labelling individuals as “SEED” to access appropriate care pathways have been raised by service users with longstanding eating disorders [12]. The STATED was developed for eating disorders across the lifespan, with guidance for pediatric populations, for clinicians to consider readiness and engagement of both the individual and their caregivers. However, a caveat was proposed by Geller and colleagues [10] that the quality-of-life focused treatment needs to be considered with care in pediatric populations given the potential for irreversible medical complications associated with the eating disorder.

The literature on severe and enduring presentations, and quality-of-life focused interventions, is almost exclusively focused on adult populations. Even with quality-of-life focused interventions, there are challenges with consensus. Mixed views have emerged in a study of clinician practices in what clinical characteristics would be most appropriate for a “quality of life” focus in the STATED. In a survey which assess clinician’s views about appropriate treatment options for young people (and their families) and adults with eating disorders, nearly half of clinician responses (48%) were not consistent with the STATED recommendations [11], suggesting that there are diverse views about who is best suited for quality-of-life focused approaches.

One of the likely factors contributing to the paucity of attention on severe and enduring presentations of eating disorders in pediatric populations is the duration of illness criterion. Although Hay and Touyz [1] proposed a three-year duration, the majority of studies in the field have required a seven-year duration of illness [2]. Given the peak incidence of eating disorders is between ages 13 to 18 years [13], it would be uncommon to see individuals in pediatric settings with a duration of illness of seven years or more. Yet, a portion of individuals with AN demonstrate symptoms emerging before age 13 years [13]. Therefore, when considering the proposed duration criteria of at least 3 years [1], it is likely that there are young people receiving clinical care in pediatric settings who would meet these proposed criteria.

The lack of consensus within the field about the definition of (and label for) severe and enduring presentations is particularly relevant when considering pediatric populations. There are concerns about potential perceptions of suggesting that eating disorder recovery is not possible for a subset of young people [14]. The term “treatment resistant AN” has been used to describe individuals (including children and adolescents) who do not respond to treatment [15]. Yet, there is risk with using this term for pediatric populations as this can imply a lack of hope for the possibility of recovery. A new term (AN-PLUS) has been proposed to describe young people with anorexia nervosa (AN) who are characterized by a poor quality of life, lack of response to evidence-based treatments, medically severe and unstable, and who are presenting with severe eating disorder symptoms [14]. Notably, there is no fixed minimum duration of illness associated with AN-PLUS. Furthermore, the terminology of AN-PLUS intentionally avoids implications about prognosis or recovery [4, 14]. A person-centred, harm reduction approach is proposed for young people with AN-PLUS in situations where the risks or harms of a treatment (including involuntary treatment options) outweigh the expected benefits [14].

The proposed AN-PLUS criteria [14] provide guidance for pediatric health professionals around the appropriateness of harm reduction approaches. Yet, with few publications on severe and enduring presentations of eating disorders in pediatric populations, it is not clear the extent to which health professionals encounter these presentations in their clinical work with pediatric populations, and to what extent they incorporate harm reduction, or quality-of-life focused approaches, or even conceptualize presentations as severe and enduring. The goals of this study were to: (1) assess the perspectives of health professionals regarding clinical care for young people with severe and enduring presentations of eating disorders, and (2) explore perceptions about appropriate treatment options for pediatric severe and enduring presentations in relation to the STATED [10] recommendations. Given the paucity of literature in the field, and the established lack of consensus about defining severe and enduring presentations [2], we anticipated clinicians would share significant barriers to providing clinical care for this population. Based on the discordance between clinical practice and the STATED recommendations that has been demonstrated for the quality-of-life focus [11], we further expected diverse clinician perspectives about treatment of young people with a severe and enduring eating disorder presentation.

## Methods

### Participants

Healthcare professionals based in Canada or Australia who provide services for young people with eating disorders were invited to participate in a two-stage online survey. We chose to limit participation to these two countries, to achieve an international perspective while considering similarities between Canada and Australia (i.e., a mix of public and privately funded health care services, and large geographical service regions). Study invitations were distributed through established networks of eating disorders health professionals in both Canada and Australia, including the Eating Disorders Association of Canada, the Australia and New Zealand Academy for Eating Disorders, the InsideOut Institute, and the British Columbia Eating Disorders Network. We briefly posted study invitations on social media of a Canadian eating disorders organization, but identified spam responses upon posting the study link, and swiftly removed the social media postings and created a new study link that was subsequently disseminated through email and professional networks. Individuals were encouraged to pass on study information to other colleagues who work with pediatric eating disorders.

English-speaking health professionals who were working in Canada or Australia, and who had provided clinical care in the past year to young people ages 25 years and under were eligible to participate. Study information letters and emails included a link to a web-based survey, which individuals could access to review consent and eligibility information. Participants reviewed a consent form online, and acknowledged understanding the study information and confirmed meeting eligibility criteria prior to proceeding to the web-based survey (Survey 1). The web-based survey and study database were maintained using Research Electronic Data Capture (REDCap; [16]), hosted by the BC Children’s Hospital Research Institute. Individuals who completed Survey 1 had the option of remaining anonymous or indicating interest in being contacted for Survey 2 (in which case, they were asked to provide their name and e-mail address). Ethics approval for this project was obtained from the University of British Columbia/Children’s and Women’s Hospital and Health Centre of British Columbia Research Ethics Board (H21-02240).

### Procedure

The goals of this study were part of a larger study on assessment of readiness/engagement in young people and their families, and clinical decision-making in relation to the STATED [10] for pediatric populations. The procedures specific to the goals relating to clinical care for severe and enduring presentations of eating disorders in young people are presented here.

Survey 1. A 25-item general survey (Survey 1) was completed by all participants, which assessed participants’ experience with providing clinical care for pediatric eating disorders. There were two questions specific to experiencing with clinical care for severe and enduring eating disorders in their practice. Participants were asked “Do you see children/adolescents with severe and enduring eating disorders in your clinical practice (e.g., individuals who have had an eating disorder for 3 or more years, and who have severe eating disorder symptoms).” A yes or no response option was provided. Participants who responded yes were then prompted with an open-ended question “What are some of the key concerns that you have in providing clinical care for children/adolescents with severe and enduring eating disorders?” We intentionally did not provide proposed criteria for severe and enduring presentations, to allow participants to evaluate whether they provide care for this population based on their own perceptions and experiences. However, drawing from Hay & Touyz [1], we provided a prompt that this may include individuals with at least a 3-year duration of illness, to ensure that participants were not responding with examples relevant to relatively short durations of illness (e.g., 12 months). The survey also included questions about participant demographics (i.e., age, gender identity, and highest level of training), in addition to clinical experience with pediatric eating disorders and work setting, which was adapted from a previous survey of health professionals from our research group [17].

Survey 2. Participants who responded to Survey 1 were asked if they wished to be contacted to participate in Survey 2, in which they review clinical vignettes and share views about treatment recommendations for pediatric eating disorders. Participants who agreed to be contacted were provided with a consent document through a study link. Participants reviewed study information, and confirmed that by completing the questionnaire they were consenting to participate in the research. A total of five vignettes were presented in Survey 2: four in which there is a recent onset of an eating disorder and youth and family readiness/engagement is manipulated across the vignettes ((1) low youth/high parents;; (2) high youth/low parents; (3) high youth/high parents; (4) low youth/low parents), and a fifth vignette with a severe and enduring eating disorder presentation with low readiness/engagement in youth and parents. These vignettes were developed by the lead author, in collaboration with a non-clinical member of the research team (SM), and piloted with five members of the research team who are involved with clinical care for eating disorders, to ensure that sufficient information was presented and that the level of readiness/engagement portrayed in the vignettes was accurately perceived. Vignettes have been demonstrated to have validity in the assessment of clinician decision-making, and recommendations for vignette development [18] were followed in the design of the vignettes used for the current study. Vignettes are available in Supplementary Materials (Appendix A). Vignettes were presented with gender-neutral pronouns and ambiguity about the gender identity of the youth, to make these scenarios inclusive across gender.

Participants were asked to identify what type of treatment they would begin with for each vignette, and whether their decision would change if the vignette presented a younger child (e.g., age 11 or 12 years). Treatment options were based on the options presented in the STATED [10]: outpatient - focus on youth/family engagement; outpatient - focus on recovery; intensive treatment service - day, inpatient, or residential with focus on recovery; outpatient with inpatient support - focus on quality of life; and hospitalization - focus on medical stabilization. “Other” (and describing) or “unsure/I don’t know” were also provided as options. Participants could select only one option. There was one open-ended question after the vignette, where participants were invited to explain their treatment choice for the vignette. There was also an open-ended question at the end of all vignettes, where participants were invited to write about their thoughts about models of care that are appropriate for young people with severe and enduring eating disorders and low readiness/engagement.

Participants who took part in the general survey were provided with the option to enter a draw for one of ten $20 gift cards (in either CAD or AUD, depending on participants’ country of residence). Contact information for those who entered the draw was stored in a separate database, to maintain the anonymity of participants. However, individuals who were interested in taking part in Survey 2 were asked to provide their e-mail address, and therefore were not able to maintain anonymity^2^. Those who took part in Survey 2 (reviewing vignettes) were offered a gift card valued at $15 (in either CAD or AUD, depending on the participant’s residence).

### Statistical analyses

Demographic data and data on experiences with providing clinical care for pediatric severe and enduring eating disorders is presented with frequencies. Chi-square analysis was performed to assess differences in the proportion of respondents who provide clinical care for severe and enduring presentations across primary, secondary and tertiary settings. The frequencies for the recommended treatment approaches for each vignette are also reported. Given the small sample size for Survey 2, analyses were performed on available data for each separate analysis. Information about the number of responses analyses were based on is provided for each variable.

Responses on the open-ended questions were coded into themes by the lead author (JSC) and second author (TP). Conventional content analysis was performed [19]. The lead author initially reviewed all open-ended responses, then re-read the responses and developed a coding scheme, which was reviewed and discussed with the second coder. Responses that clustered together were grouped under a theme. A total of nine themes were initially identified, which were then reviewed and reduced based on responses that were commonly occurring together. The second coder independently coded all responses. The lead author is a clinical psychologist and researcher with 20 years of experience in the field of eating disorders, and the second author is a bachelor’s level research coordinator with 18 months of experience in a specialized eating disorders research setting. The agreement between coders was reasonable, with Κ ranging from 0.66 to 0.83 for Survey 1, and 0.63–1.00 for Survey 2. After determining the agreement between coders, the two coders met to review and resolve inconsistent responses, and mutually agreed on the final coding scheme and ratings for open-ended responses. Experienced eating disorders clinicians with expertise in adolescent medicine (PYL) and child psychiatry (SG), as well as clinical ethicists with experience supporting clinical decision-making in pediatric eating disorders (AV, AOP) collaborated on the interpretation of results.

## Results

### Participants

A total of 120 individuals entered the study link and provided consent. Of these, 10 responses were identified as spam and were removed from the database (responses immediately after posting on social media, which all included illogical responses), and an additional 10 responses contained no responses to study questions. A total of 100 individuals provided consent for the study and went on to complete at least some items on Survey 1. Of these, 85 participants completed the questions related to clinical care for severe and enduring eating disorders. Data from these 85 participants are presented. There were 61 participants living in Canada (71.8%), 23 in Australia (27.1%), and 1 participant who did not report their country of residence (1.2%). Respondents included a mixture of physicians (*n* = 17, 20%), nursing staff (*n* = 10, 11.8%, and allied health professionals (*n* = 58, 68.2%). Participant demographic information is presented in Table 1.

**Table 1:** Participant demographics

	Sample size of health professionals (*n* = 85)
Age	
25–34 years	35 (41.2%)
35–44 years	30 (35.3%)
45–54 years	15 (17.6%)
55–64 years	3 (3.5%)
Missing	2 (2.4%)
**Gender Identity**	
Male	6 (7.1%)
Female	73 (85.9%)
Non- Binary	1 (1.2%)
None of the above Missing	1 (1.2%) 4 (4.7%)
**Years working the field of ED**	
< 1	12 (14%)
1–4	31 (36.5%)
5–9	20 (23.5%)
10–14	13 (15.3%)
15–19	3 (3.5%)
20+ Missing	5 (5.9%) 1 (1.2%)
**Primary Work Setting**	
Private Practice / Primary Care	14 (16.5%)
Secondary Services	33 (38.8%)
Tertiary Services Missing	37 (43.5%) 1 (1.2%)
**Work Setting**	
Rural	7 (8.2%)
Suburban	22 (25.9%)
Urban Missing	55 (64.7%) 1 (1.2%)
**Primary Treated Age Group** ^a^	
Children/Adolescents	28 (32.9%)
Youth/Adults	11 (12.9%)
Across the lifespan	46 (54.1%)

^a^Children/adolescents were defined as age 18 years and under. Youth/adults were defined as 17 years and above. Respondents were able to select more than one age group. If respondents worked with both children/adolescents and adults, they were added to ‘across the lifespan’ category. The overlapping categories were included given that some programs in the region offer support to transition-age youth (i.e., who are transitioning out of the pediatric system, into the adult system)

Sixty respondents to Survey 1 expressed interest in taking part in Survey 2, of whom 40 responded to the invitation that was distributed and were sent a study link. A total of 25 individuals responded to at least some of Survey 2, and 20 respondents completed this follow-up survey. Data from all 25 respondents was maintained for analyses, and available data for each variable is presented.

### Experiences in providing care for pediatric severe and enduring presentations

The majority of respondents (*n* = 70, 82.4%) indicated that they provide clinical care for young people with severe and enduring eating disorder presentations. A follow-up chi-square analysis demonstrated that those working in tertiary (hospital-based) settings were more likely to work with severe and enduring presentations (*n* = 35/37, 94.6%) in comparison to those in secondary (community-based) programs (*n* = 24/33, 72.7%) or primary practice/private practice (*n* = 10/14, 71.4%), χ^2^ (2, *N* = 84) = 7.00, *p* = .03. However, it is noteworthy that across settings, the majority of health professionals report providing clinical care for severe and enduring presentations in young people.

Of the 70 individuals who reported providing clinical care for pediatric severe and enduring presentations, 59 provided an open-ended response to highlight their key concerns about clinical care with this population. There was a wide range of details that respondents shared in the open-ended responses, with the word count of responses ranging from 2 to 494 (mean = 39.3 words; median = 21 words). A total of 6 themes were constructed from the responses. The total number of responses that were coded under each of the themes is noted (given that some responses were lengthy, many were coded under more than one theme).

*Medical management, transitions in care, and supporting concurrent psychiatric diagnoses* (*n* = 29): Participants highlighted medical monitoring and stability, access to medical care, as well as managing psychiatric comorbidities as key concerns. Participants also shared challenges in supporting both an eating disorder and other mental health diagnosis, indicating that limited access to paediatric psychiatrists and other specialized health professionals means that “access to appropriate pharmacological treatment with adequate monitoring is more difficult to access and clients [don’t] have the full benefit of psychological treatment” (participant 75).

Some responses bridged both resources and medical/psychiatric management themes, indicating that there is “Not sufficient resources to provide outpatient treatment and not sufficient community services and ability to treat comorbidities” (participant 91). Responses also highlighted challenges with medical monitoring and other clinical care in the context of transitions in care between the pediatric and adult systems. One respondent shared a concern surrounding “the capacity of the primary care provider to continue to follow the client medically, ESPECIALLY youth that are 17 /18 and aging out of pediatric medical care systems or who are 18 + and aging out of provincial mental health systems” (participant 39).

*Availability of resources or appropriate treatment options (n* = 28): Participants shared their perspectives about the “lack of resources for this population” (participant 35). Many participants also highlighted the lack of diverse services in their area, with significant geographical barriers (e.g., more than 4 h drive to intensive treatment service options). Barriers in rural and remote settings were also identified, with a lack of dedicated clinicians who are trained in supporting eating disorders, or limited resources in the local eating disorder program. Several respondents highlighted lack of alternatives to family-based therapy (FBT) in their setting, or shared concerns about the quality of evidence-based approaches (e.g., FBT that did not adhere to the model). “There’s no other modality to offer in our community if FBT doesn’t work” (participant 21). Another participant reflected on the need to train clinicians in a variety of approaches, highlighting:

Clinicians need to be upskilled as to when to choose FBT versus say parent-supported CBT-ED [cognitive behavioural therapy for eating disorders], or AFT [adolescent focused therapy], or MFT [multi-family therapy]. Yes, FBT is the leading EBP Tx [evidence-based practice treatment], but what happens when FBT is not a right fit? (participant 95).

*Burnout and supports needed for caregivers and clinicians* (*n* = 23): Participants shared concerns about limited supports that are available both to parents/caregivers of young people with eating disorders, as well as staff who are providing clinical care. Respondents indicated that there is a lack of resources, including respite, for parents supporting their child with a severe and enduring presentation. Further, the limited resources available to parents/caregivers was perceived to impact their ability to continue to provide the level of care needed (e.g., meal support and emotional support). Several respondents shared experiences with limited or no family involvement. Clinician burnout and lack of supports (including access to appropriate education and training) was also highlighted. Respondents also shared concerns about clinician moral distress, compassion fatigue, and apathy. Participants presented key concerns about both “parent burnout/fatigue” and “staff fatigue” (participant 80) in the same response.

*Evidence-based treatments and treatment experiences* (*n* = 22): Respondents shared that there is “a lack of clear evidence-based guidelines for the treatment of these youth” (participant 11). The lack of alternatives to FBT or intensive (e.g., inpatient) treatment were raised, in addition to concerns about past treatment experiences that “did more harm than good” (participant 36). Several participants highlighted challenges with the readiness and willingness of young people with severe and enduring presentations to participate in treatment, and how to provide adequate care to engage these individuals in treatment.

*Ethical considerations and trauma-informed practice* (*n* = 11): Several participants raised ethical considerations, including those related to involuntary admissions, and considerations around trauma-informed practice and providing safe and appropriate clinical care. One participant raised concerns about labelling young people as having “SEED” and whether it is ethical to treat them in the same way as adults, questioning “should we always be recovery-focused given their age and our role as adults to guide them during their development?” (participant 59). Participants shared that young peoples’ experiences in treatment may be experienced as traumatic, including physical restraint to provide nasogastric tube feeding, indicating that the “re-traumatisation of children and adolescents is therefore my primary concern” (participant 81).

*Institutionalization, community connections, and supporting safety-related concerns in treatment* (*n* = 9): Respondents shared concerns about the risks associated with long-term hospitalization on development, and engagement in school, family, and vocational interests. Some participants also shared concerns about how to safely contain behaviour in treatment settings: “will having consequences/boundaries/expectations lead to dysregulation that we cannot contain?” (participant 7).

#### Survey 2: Reflections on Case vignettes

Participants who took part in Survey 2 were asked to identify the treatment option that they would recommend to begin with for each vignette, and the proportion of respondents selecting each treatment option was assessed. There was the highest consensus amongst participants for cases 2 (high youth/low parent readiness/engagement), 3 (high youth/high parent readiness/engagement), and 4 (low youth/low parent readiness/engagement), with more than 75% of respondents selecting the same treatment recommendation for these vignettes. In contrast, 56% of respondents chose the same treatment recommendation for vignette 1 (low youth/high parent readiness/engagement). Vignette 5 (low youth/low parent readiness/engagement with severe and enduring presentation) presented with the lowest consensus of all vignettes (*≤* 35% of participants chose the same recommendation). See Table 2 for treatment options selected by respondents for each of the vignettes (note that the relative proportions for each vignette varies as there was a range of 20–25 responses across vignettes).

**Table 2:** Participant responses indicating the type of treatment that they would recommend for each of five vignettes, in which youth and parent readiness/engagement is adjusted across vignettes, in addition to duration of illness/treatment history for case 5

	Case 1 ↓youth ↑ parent (*n* = 25)	Case 2 ↑youth ↓ parent (*n* = 21)	Case 3 ↑youth ↑parent (*n* = 21)	Case 4 ↓youth ↓parent (*n* = 20)	Case 5 ↓youth ↓parent (*n* = 20)
Outpatient (focus on engagement)	44% (*n* = 11)	76% (*n* = 16)	19% (*n* = 4)	85% (*n* = 17)	20% (*n* = 4)
Outpatient (focus on recovery)	56% (*n* = 14)	19% (*n* = 4)	76% (*n* = 16)	15% (*n* = 3)	0% (*n* = 0)
Intensive Treatment (recovery focus)	0% (*n* = 0)	5% (*n* = 1)	5% (*n* = 1)	0% (*n* = 0)	25% (*n* = 5)
Outpatient with inpatient support (quality of life focus)	0% (*n* = 0)	0% (*n* = 0)	0% (*n* = 0)	0% (*n* = 0)	35% (*n* = 7)
Hospitalization (focus on medical stabilization)	0% (*n* = 0)	0% (*n* = 0)	0% (*n* = 0)	0% (*n* = 0)	5% (*n* = 1)
Other	0% (*n* = 0)	0% (*n* = 0)	0% (*n* = 0)	0% (*n* = 0)	15% (*n* = 3)

Participants’ responses to the vignette with the severe and enduring presentation were classified as STATED-consistent if they selected outpatient with inpatient support (focus on quality of life) [10]. One of the “other” responses was recorded to outpatient with inpatient support (focus on quality of life), bringing the total to 8 of the 20 respondents (40%) who shared a treatment recommendation that aligned with the STATED [10] recommendations.

The majority of respondents (*n* = 13/20, 65%) indicated that their treatment choice would have differed if the vignette presented a younger individual (e.g., age 11 or 12 years). Open-ended responses were evaluated to contextualize the treatment choice indicated by participants (a total of 17 responses out of the 20 participants who responded to vignette 5, with the word count ranging between 7 and 167 words; mean of 31.3 and median of 21 words). Even with the 16-year-old featured in the vignette, there was clear discomfort about the potential for a quality-of-life focus in participants’ explanations. One participant shared:

This is a difficult case scenario as it seems completely bizarre to offer quality of life supports to a youth. … I am unsure if this is the appropriate method of action due to Spencer being only a youth and not an adult however I do not see the benefit in more intensive treatment or hospitalization for the eating disorder” (participant 14).

Four themes were constructed from the responses about treatment choice: (1) reflections on adult models of care and alternative treatment goals (*n* = 7); (2) engagement with young people (*n* = 6); (3) parent support/focus (*n* = 5) and (4) a full recovery focus (*n* = 3). The participants who emphasized a recovery focus expressed concerns about it being premature to shift to a focus on quality of life, and recommended engagement with the young person, or considering the option of day treatment, as well as providing clinical care to parents/caregivers. One participant shared the perspective that recovery-focused treatment could remain a goal, while taking a “break” to focus on quality of life with the young person and parents while establishing non-negotiables (participant 19).

Open-ended responses to a question about models of care for severe and enduring presentations were evaluated, and there was a range in participants’ engagement on this question (total of 18 responses out of the 20 participants who completed all vignettes, with responses ranging from 1 to 157 words; mean of 49 and median of 37 words). Three themes were constructed from these responses: challenges with existing models/lack of research (*n* = 7); engagement in young people vs. caregivers (*n* = 7); and the importance of recognizing the potential for recovery (*n* = 7). Respondents were divided between the potential utility of alternative models focusing on quality of life for young people, and the importance of remaining recovery-focused in pediatric settings. Participants who endorsed the importance of a recovery-focused model/approach highlighted ongoing brain development through adolescence, and shared recommendations for ongoing assessment of readiness. One participant suggested that it may be possible to support young people “in the least invasive manner, while maintaining safety and working with caregivers to improve longer term change” (participant 11). Caveats to alternative models were raised, including one participant who shared that “first we must try usual treatment approaches for many years, and have reached real re[-]nutrition periods before considering it did not work…” (participant 22). Six participants shared uncertainty about the most appropriate models of care, with comments indicating that it is currently “ad hoc and unsatisfactory” (participant 5), or that the question about models of care for pediatric severe and enduring presentations was “a really tough one, and I really don’t know” (participant 15).

## Discussion

The results of this study provide a preliminary indication that clinicians are caring for young people with severe and enduring presentations of eating disorders. Over 80% of study participants, regardless of their work setting, shared that they have provided clinical care for young people with severe and enduring presentations of eating disorders, though it was more common for hospital-based clinicians than for clinicians in primary or community-based services. As expected, clinicians shared significant challenges with providing clinical care for this population. The main themes identified by participants were lack of resources, and limited availability of a variety of models of care, as well as concerns about burnout both in parents/caregivers and clinical staff. Participants also highlighted ethical concerns about identifying young people as having a severe and enduring presentation, and how to safely and appropriately provide care for both young people and their families. Themes of limited resources and carer burden aligned with previous reports of clinicians working in adult settings [20], reflecting the complex care needs of this population across the lifespan.

We expected diverse clinician perspectives about treatment of young people with a severe and enduring eating disorder presentation, given previously reported inconsistencies in clinical practice in relation to the STATED [11]. Responses on Survey 2 demonstrated the lowest level of participant agreement for the vignette with a severe and enduring presentation, in contrast with the clinical vignettes with a shorter duration of illness (including a vignette where there was low readiness for both youth and parent, but with a shorter duration of illness). Less than half of participant responses for the vignette with a severe and enduring presentation aligned with the STATED recommendations for a model of care focused on quality of life [10]. Open-ended responses further demonstrated a division in participant responses about the most appropriate models of care for severe and enduring presentations in young people, with some participants endorsing the potential utility of a quality-of-life focus, while a small group of participants stressed the importance of continuing to focus on recovery.

Several participants expressed concerns about labelling young people as having severe and enduring eating disorders. This aligns with published recommendations based on engagement with service users about the importance of treatment pathways focused on quality of life not resulting in individuals being labelled as “SEED” [12] or “treatment resistant”. One of the advantages of using the STATED [10] to guide clinical decision-making about treatment pathways is that it is multidimensional, with three patient/family characteristics used to guide treatment planning (medical stability, symptom severity/life interference, and readiness/engagement). Thus, the pathway for STATED [10]allows the clinician to introduce a quality-of-life focus in a way that could feel less judgemental to young people and their families about their future potential. Future research that engages with individuals with lived/living experience about the application of STATED for young people with eating disorders is needed.

Based on the reflections shared by clinicians who participated in the current study, it appears that pediatric service providers are aware of emerging models of care for severe and enduring presentations. Yet, they also shared ethical concerns about the appropriateness of these services for pediatric populations, and there was uncertainty about the most appropriate models of care expressed by one-third of participants in Survey 2. Some of the discomfort expressed by clinicians was not specific to severe and enduring eating disorders, but rather challenges in the system of care (e.g., timely access to services, clinician availability, provision of appropriate evidence-based approaches) that was also perceived to be connected the development of severe and enduring presentations. There was a suggestion that taking a quality-of-life approach might be akin to compromising with the youth or giving up. However, taking a dialectic view in which recovery-focused and quality-of-life approaches are not in opposition may help address some of the discomfort expressed by clinicians. As suggested by a participant, it may be appropriate to shift to quality-of-life focused approaches as a break after attempting evidence-based, recovery-focused approaches, while continually re-assessing next steps and continuing to hold the ultimate goal of recovery. Furthermore, clinicians’ awareness of alternatives to FBT, and the ability to provide other evidence-based approaches, is an important aspect of care. Although FBT is the only approach that is strongly recommended by the Canadian pediatric eating disorder guidelines, several other approaches can be recommended (e.g., cognitive behavioural therapy, adolescent focused psychotherapy, adjunctive yoga and atypical antipsychotics) [21].

Ethics support for clinicians, particularly direct care staff working in hospital settings, is a key service recommendation for pediatric presentations of AN-PLUS [14], and aligns with themes captured in the current study. Part of the challenge in working with young people with eating disorders is the question of capacity of making decisions, particularly in cases where the young person has limited or no interest in engaging in treatment. Adolescents may still be developing cognitive skills to make decisions, as well as their own value system and identity, and the potential influence of the illness on values and sense of self. From an ethical perspective, it is important to consider the relational nature of autonomy and decision making. At times, individuals may make choices beyond individual interests, and their values are influenced by their relationships and environment (see e.g [22]). Supporting clinicians to navigate ethical considerations in the unique context of pediatric care, where relational autonomy is distinctly explicit can alleviate the moral distress that arises with these complex clinical presentations. Similarly, collaborative care, and fostering compassion for others in clinicians in the context of severe and enduring presentations can also be helpful to clinicians [23].

The responses around the need for family/caregiver-focused support that were shared in the survey also highlights a major gap in existing research in the area, as there are a currently no interventions that have been developed and evaluated for caregivers of young people with a severe and enduring presentation. The New Maudsley Collaborative Care approach was developed to support carers of adults with a broad range of eating disorders [24]. The New Maudsley method has been adapted into a brief, 2-session workshop, which was effective in improving carer burden and self-efficacy relative to a waitlist control group [25]. The New Maudsley method has also inspired a skills-based training for parents of adolescents with eating disorders, with emerging evidence for the utility of this training in improving parental skills and reducing guilt and stress [26]. The need to further develop caregiver-focused interventions beyond FBT has been identified by parents whose child’s distress persisted after FBT [27].

In the surveys, we intentionally did not provide specific criteria to define severe and enduring presentations of pediatric eating disorders (given the lack of definition in wide use for pediatric populations). However, we did provide a prompt suggesting a minimum duration of three years based on the criteria put forth by Hay and Touyz [1]. The lack of specific definition provided to participants has a potential advantage in ensuring we could capture broad responses about clinician perceptions about this population, while also a limitation in the variability of how clinicians may have conceptualized a severe and enduring presentation. Future research will benefit from using the newly developed AN-PLUS criteria [14], and assessing the frequency with which clinicians are supporting these presentations. It is notable in the proposed AN-PLUS criteria for pediatric eating disorders [14], there is not a duration component. Instead, there is a focus on engagement in evidence-based treatments without improvement, in addition to medical stability, quality of life, and symptom severity criteria.

One of the strengths of this study is the engagement of a sample of clinicians who are based in a variety of clinical settings. Furthermore, the study had a broad focus on perspectives of health professionals on clinical care for pediatric eating disorders, and therefore was not specifically advertised as relating to severe and enduring presentations. Therefore, the likelihood of biased responses of those with a particular interest in this area was mitigated. However, there were limited responses to Survey 2, including some participants who discontinued participation prior to reaching the vignette with the severe and enduring presentation (given that the vignettes were not presented in a random order), which may impact of the generalizability of study results. This initial exploratory study relied primarily on descriptive analyses, and we did not have sufficient power to compare the likelihood of treatment recommendations across the vignettes, or examine clinician characteristics that were associated with positive views of quality-of-life focused approaches. Future research aimed at consensus building in interested parties, including clinicians, young people, and caregivers with lived experience is warranted.

## Conclusions

The current study provides preliminary evidence that a high proportion of pediatric eating disorder clinicians report caring for severe and enduring presentations in the pediatric population. Although clinicians shared their perspectives about the potential utility of quality-of-life focused interventions for severe and enduring presentations, there were significant concerns about the appropriateness of these interventions for young people with eating disorders raised by a portion of participants. Less than half of clinicians shared treatment recommendations that aligned with the STATED recommendations [10], for a vignette depicting a severe and enduring presentation of a pediatric eating disorder. Criteria for young people with AN who would benefit from a quality-of-life focus have been proposed [14]. Further research to build consensus across clinicians, young people and families about when and how to integrate a quality-of-life focus is much needed.

### Electronic supplementary material

Below is the link to the electronic supplementary material.

Supplementary Material 1

## Data Availability

The data that support the findings of this study are available from the corresponding author upon reasonable request.

## Footnotes

1 We use the term “persistent” in the title of this paper in lieu of “enduring” to highlight our belief that eating disorder recovery is possible, regardless of duration of illness or past treatment experiences. However, hereafter we continue using the term “severe and enduring” in the paper, except where citing a paper where authors use a different term, as this is the predominant terminology in the field, and has been identified by individuals with lived experience as preferable over other labels such as “chronic” [3].

2 Ethics approval was obtained to link the responses between the Survey 1 and Survey 2. Unfortunately, an error in the REDCap survey distribution set-up meant that a portion (40%) of participant responses to Survey 2 were recorded anonymously, thereby precluding the possibility of linking participant demographics with responses to the vignettes for these cases.
