# Supplementary material for: Clinical care for severe and persistent eating disorders in pediatric populations: Perspectives of health professionals
Source: J Eat Disord. 2024 Jun 17;12:83. doi: 10.1186/s40337-024-01044-6 (PMC11181587; doi:10.1186/s40337-024-01044-6)
Supplement: Supplementary file 1 — Supplementary Material 1 [file 40337_2024_1044_MOESM1_ESM.docx]

Appendix A: Vignette Descriptions

Gender neutral pronouns are used in the following examples.

Assume that there are no limitations for either the system or the family in the following scenarios (i.e., waitlists, parental financial concerns, etc).

There is repetition in some information across cases – the information that is the same in each vignette is presented in a box.

**Case 1 (low youth/high parent readiness)**

A primary care physician referred Spencer (age 16 years) to a specialized eating disorders program due to concerns regarding Spencer’s eating habits and weight. At the assessment, Spencer and their parents shared that Spencer started focusing on healthy eating a year ago, and since then their eating has become increasingly restrictive. Weight and height were measured at assessment, and Spencer was below the 3^rd^ centile for Body Mass Index (BMI) for their sex/age. Based on information about Spencer’s height and weight provided by parents, Spencer’s weight had previously tracked along the 50^th^ centile for BMI. Spencer is around 78% of treatment goal weight. Spencer reports significant body image concerns, and worries about substantial weight gain if they don’t continue their daily exercise routine. Spencer took part in a medical assessment with a pediatrician, and did not present with any signs of medical instability (e.g., heart rate, bloodwork results). Spencer presents with symptoms of generalized anxiety, but has never received any formal psychiatric diagnoses or had any previous treatment for mental health concerns. Spencer denies suicidal ideation or self-harm behaviours, and the family denies and current or past history of violence or neglect. Spencer is given a diagnosis of anorexia nervosa – restrictive subtype by the team.

Spencer’s parents are very concerned about Spencer, and express that they are willing to do whatever is necessary to support Spencer to return to a healthy weight range and improve Spencer’s eating habits. They share that they have sought out resources on supporting a child with an eating disorder, and have started to increase the amount of food they are providing to Spencer. Spencer says that the doctors and health professionals are over-reacting, and that they are just focused on healthy eating like many other teenagers at their school. Spencer indicates that they don’t think they need treatment, and don’t agree that they have an eating disorder.

What type of treatment would you begin with for Spencer and their family?

☐Outpatient (focus on youth/parent engagement) (if this option is selected, please indicate with whom the engagement effort would focus)

☐Outpatient (focus on recovery)

☐Intensive treatment services (day treatment, inpatient or residential; focus on recovery)

☐Outpatient with inpatient support (focus on quality of life)

☐Hospitalization (focus on medical stabilization)

☐Other (please describe)

☐Unsure/I don’t know

Please rate how appropriate you feel that your choice would be for Spencer and their family:

“definitely not appropriate” (score of 1–2), “probably not appropriate” (score of 3–4), “probably appropriate” (score of 5–6), “appropriate” (score of 7–8), and “definitely necessary” (score of 9).

If outpatient: What therapeutic approach would you recommend? (please select your primary recommendation, and provide your reasons for this choice)

How did you come to make this decision about the best treatment fit? (e.g., what were some of the features of the vignette that made you select this treatment option)

To what extent does this vignette portray a clinical situation that you have encountered in your clinical practice?

Not at all 1 2 3 4 5 Very Much

Would your treatment choice have differed if the vignette presented a younger individual (e.g., age 11 or 12)?

Yes No (if yes – please explain)

Would your treatment choice have differed if the vignette presented additional concerns with co-occurring mental health concerns (e.g., if Spencer also reported low mood and occasional non-suicidal self-injurious behavior)?

Yes No (if yes – please explain)

**Case 2 (high youth/low parent readiness)**

A primary care physician referred Spencer (age 16 years) to a specialized eating disorders program due to concerns regarding Spencer’s eating habits and weight. At the assessment, Spencer and their parents shared that Spencer started focusing on healthy eating a year ago, and since then their eating has become increasingly restrictive. Weight and height was measured at assessment, and Spencer was below the 3^rd^ centile for Body Mass Index (BMI) for their sex/age. Based on information about Spencer’s height and weight provided by parents, Spencer’s weight had previously tracked along the 50^th^ centile for BMI. Spencer is around 78% of treatment goal weight. Spencer reports significant body image concerns, and worries about substantial weight gain if they don’t continue their daily exercise routine. Spencer took part in a medical assessment with a pediatrician, and did not present with any signs of medical instability (e.g., heart rate, bloodwork results). Spencer presents with symptoms of generalized anxiety, but has never received any formal psychiatric diagnoses or had any previous treatment for mental health concerns. Spencer denies suicidal ideation or self-harm behaviours, and the family denies and current or past history of violence or neglect. Spencer’s is given a diagnosis of anorexia nervosa – restrictive subtype by the team.

Spencer’s parents share their view that it is up to their child to decide to make changes to their eating/weight, and that they do not feel their participation in therapy would be helpful. They indicate that they can help support bringing Spencer to medical and therapy appointments. Spencer expresses that they are bothered by physical symptoms that have been getting worse (e.g., dizziness, hair loss), and shares that although they are scared about starting treatment, they recognize that they need support.

What type of treatment would you begin with for Spencer and their family?

☐Outpatient (focus on youth/parent engagement) (if this option is selected, please indicate with whom the engagement effort would focus)

☐Outpatient (focus on recovery)

☐Intensive treatment services (day treatment, inpatient or residential; focus on recovery)

☐Outpatient with inpatient support (focus on quality of life)

☐Hospitalization (focus on medical stabilization)

☐Other (please describe)

☐Unsure/I don’t know

Please rate how appropriate you feel that your choice would be for Spencer and their family:

“definitely not appropriate” (score of 1–2), “probably not appropriate” (score of 3–4), “probably appropriate” (score of 5–6), “appropriate” (score of 7–8), and “definitely necessary” (score of 9).

If outpatient: What therapeutic approach would you recommend? (please select your primary recommendation, and provide your reasons for this choice)

How did you come to make this decision about the best treatment fit? (e.g., what were some of the features of the vignette that made you select this treatment option)

To what extent does this vignette portray a clinical situation that you have encountered in your clinical practice?

Not at all 1 2 3 4 5 Very Much

Would your treatment choice have differed if the vignette presented a younger individual (e.g., age 11 or 12)?

Yes No (if yes – please explain)

Would your treatment choice have differed if the vignette presented additional concerns with co-occurring mental health concerns (e.g., if Spencer also reported low mood and occasional non-suicidal self-injurious behavior)?

Yes No (if yes – please explain)

**Case 3 (high youth/high parent readiness)**

A primary care physician referred Spencer (age 16 years) to a specialized eating disorders program due to concerns regarding Spencer’s eating habits and weight. At the assessment, Spencer and their parents shared that Spencer started focusing on healthy eating a year ago, and since then their eating has become increasingly restrictive. Weight and height were measured at assessment, and Spencer was below the 3^rd^ centile for Body Mass Index (BMI) for their sex/age. Based on information about Spencer’s height and weight provided by parents, Spencer’s weight had previously tracked along the 50^th^ centile for BMI. Spencer is around 78% of treatment goal weight. Spencer reports significant body image concerns, and worries about substantial weight gain if they don’t continue their daily exercise routine. Spencer took part in a medical assessment with a pediatrician, and did not present with any signs of medical instability (e.g., heart rate, bloodwork results). Spencer presents with symptoms of generalized anxiety, but has never received any formal psychiatric diagnoses or had any previous treatment for mental health concerns. Spencer denies suicidal ideation or self-harm behaviours, and the family denies and current or past history of violence or neglect. Spencer is given a diagnosis of anorexia nervosa – restrictive subtype by the team.

Spencer’s parents are very concerned about Spencer, and express that they are willing to do whatever is necessary to support Spencer to return to a healthy weight range and improve Spencer’s eating habits. They share that they have sought out resources on supporting a child with an eating disorder, and have started to increase the amount of food they are providing to Spencer. Spencer expresses that they are bothered by physical symptoms that have been getting worse (e.g., dizziness, hair loss), and shares that although they are scared about starting treatment, they recognize that they need support.

What type of treatment would you begin with for Spencer and their family?

☐Outpatient (focus on youth/parent engagement) (if this option is selected, please indicate with whom the engagement effort would focus)

☐Outpatient (focus on recovery)

☐Intensive treatment services (day treatment, inpatient or residential; focus on recovery)

☐Outpatient with inpatient support (focus on quality of life)

☐Hospitalization (focus on medical stabilization)

☐Other (please describe)

☐Unsure/I don’t know

Please rate how appropriate you feel that your choice would be for Spencer and their family:

“definitely not appropriate” (score of 1–2), “probably not appropriate” (score of 3–4), “probably appropriate” (score of 5–6), “appropriate” (score of 7–8), and “definitely necessary” (score of 9).

If outpatient: What therapeutic approach would you recommend? (please select your primary recommendation, and provide your reasons for this choice)

How did you come to make this decision about the best treatment fit? (e.g., what were some of the features of the vignette that made you select this treatment option)

To what extent does this vignette portray a clinical situation that you have encountered in your clinical practice?

Not at all 1 2 3 4 5 Very Much

Would your treatment choice have differed if the vignette presented a younger individual (e.g., age 11 or 12)?

Yes No (if yes – please explain)

Would your treatment choice have differed if the vignette presented additional concerns with co-occurring mental health concerns (e.g., if Spencer also reported low mood and occasional non-suicidal self-injurious behavior)?

Yes No (if yes – please explain)

**Case 4 (low youth/low parent readiness)**

A primary care physician referred Spencer (age 16 years) to a specialized eating disorders program due to concerns regarding Spencer’s eating habits and weight. At the assessment, Spencer and their parents shared that Spencer started focusing on healthy eating a year ago, and since then their eating has become increasingly restrictive. Weight and height were measured at assessment, and Spencer was below the 3^rd^ centile for Body Mass Index (BMI) for their sex/age. Based on information about Spencer’s height and weight provided by parents, Spencer’s weight had previously tracked along the 50^th^ centile for BMI. Spencer is around 78% of treatment goal weight. Spencer reports significant body image concerns, and worries about substantial weight gain if they don’t continue their daily exercise routine. Spencer took part in a medical assessment with a pediatrician, and did not present with any signs of medical instability (e.g., heart rate, bloodwork results). Spencer presents with symptoms of generalized anxiety, but has never received any formal psychiatric diagnoses or had any previous treatment for mental health concerns. Spencer denies suicidal ideation or self-harm behaviours, and the family denies and current or past history of violence or neglect. Spencer is given a diagnosis of anorexia nervosa – restrictive subtype by the team.

Spencer’s parents share their view that it is up to their child to decide to make changes to their eating/weight, and that they do not feel their participation in therapy would be helpful. They indicate that they can help support bringing Spencer to medical and therapy appointments. Spencer says that the doctors and health professionals are over-reacting, and that they are just focused on healthy eating like many other teenagers at their school. Spencer indicates that they don’t think they need treatment, and don’t agree that they have an eating disorder.

What type of treatment would you begin with for Spencer and their family?

☐Outpatient (focus on youth/parent engagement) (if this option is selected, please indicate with whom the engagement effort would focus)

☐Outpatient (focus on recovery)

☐Intensive treatment services (day treatment, inpatient or residential; focus on recovery)

☐Outpatient with inpatient support (focus on quality of life)

☐Hospitalization (focus on medical stabilization)

☐Other (please describe)

☐Unsure/I don’t know

Please rate how appropriate you feel that your choice would be for Spencer and their family:

“definitely not appropriate” (score of 1–2), “probably not appropriate” (score of 3–4), “probably appropriate” (score of 5–6), “appropriate” (score of 7–8), and “definitely necessary” (score of 9).

If outpatient: What therapeutic approach would you recommend? (please select your primary recommendation, and provide your reasons for this choice)

How did you come to make this decision about the best treatment fit? (e.g., what were some of the features of the vignette that made you select this treatment option)

To what extent does this vignette portray a clinical situation that you have encountered in your clinical practice?

Not at all 1 2 3 4 5 Very Much

Would your treatment choice have differed if the vignette presented a younger individual (e.g., age 11 or 12)?

Yes No (if yes – please explain)

Would your treatment choice have differed if the vignette presented additional concerns with co-occurring mental health concerns (e.g., if Spencer also reported low mood and occasional non-suicidal self-injurious behavior)?

Yes No (if yes – please explain)

**Case 5 (low youth/low parent readiness, longstanding/persistent presentation)**

Spencer (age 16 years) was first referred to a specialized eating disorders program four years ago. At that time, Spencer was diagnosed with anorexia nervosa-restrictive subtype, and started family-based therapy. Spencer did not make progress in family-based therapy, and stepped up to the day treatment program, which they attended for 3 months and were then discharged back to outpatient support. Over the past three years, Spencer has been hospitalized multiple times for medical instability (low heart rate), and has been admitted twice to an intensive inpatient eating disorders program. Spencer typically gains weight during the inpatient admissions, but loses this weight rapidly after discharge. Spencer has recently started engaging in binge-eating and vomiting episodes, and has been losing weight. They are being seen in an outpatient program in a specialized eating disorders service. Spencer does not yet meet criteria for hospitalization for medical instability, but the physicians are concerned about the possibility of medical instability given recent vital signs and bloodwork results. Spencer and their parents attend a meeting with the team to re-assess treatment options. At this meeting, Spencer let the team know that they do not want to gain weight and are not interested in meeting with a therapist or starting a more intensive treatment. Parents share with the team that they are both very worried about Spencer, yet are exhausted and feeling ‘stuck’ and don’t know what they can do to shift Spencer’s thinking and behavior.

What type of treatment would you proceed with for Spencer and their family?

☐Outpatient (focus on youth/parent engagement) (if this option is selected, please indicate with whom the engagement effort would focus)

☐Outpatient (focus on recovery)

☐Intensive treatment services (day treatment, inpatient or residential; focus on recovery)

☐Outpatient with inpatient support (focus on quality of life)

☐Hospitalization (focus on medical stabilization)

☐Other (please describe)

☐Unsure/I don’t know

Please rate how appropriate you feel that your choice would be for Spencer and their family:

“definitely not appropriate” (score of 1–2), “probably not appropriate” (score of 3–4), “probably appropriate” (score of 5–6), “appropriate” (score of 7–8), and “definitely necessary” (score of 9).

If outpatient: What therapeutic approach would you recommend? (please select your primary recommendation, and provide your reasons for this choice)

How did you come to make this decision about the best treatment fit? (e.g., what were some of the features of the vignette that made you select this treatment option)

To what extent does this vignette portray a clinical situation that you have encountered in your clinical practice?

Not at all 1 2 3 4 5 Very Much

**Open-ended questions:**

Hoes does readiness/engagement of the youth factor into your treatment decisions?

How does readiness/engagement of the parents/family factor into your treatment decisions?

How does co-occurring mental health symptomatology factor into your treatment decisions?

What are your views about incorporating assessment of readiness/engagement of youth and their families? Please describe factors that may help or hinder this process.

What are your thoughts about models of care that may be helpful for youth with severe and enduring eating disorders and low readiness/engagement?

Are there any additional thoughts/comments you would like to share regarding treatment for pediatric eating disorders?
